# Oxidative DNA Damage Accelerates Skin Inflammation in Pristane-Induced Lupus Model

**DOI:** 10.3389/fimmu.2020.554725

**Published:** 2020-09-24

**Authors:** Gantsetseg Tumurkhuu, Shuang Chen, Erica N. Montano, Duygu Ercan Laguna, Gabriela De Los Santos, Jeong Min Yu, Malcolm Lane, Michifumi Yamashita, Janet L. Markman, Luz P. Blanco, Mariana J. Kaplan, Kenichi Shimada, Timothy R. Crother, Mariko Ishimori, Daniel J. Wallace, Caroline A. Jefferies, Moshe Arditi

**Affiliations:** ^1^Division of Rheumatology, Department of Medicine, Cedars-Sinai Medical Center, Los Angeles, CA, United States; ^2^Division of Pediatric Infectious Diseases and Immunology, Cedars-Sinai Medical Center, Los Angeles, CA, United States; ^3^Department of Biomedical Sciences, Cedars-Sinai Medical Center, Los Angeles, CA, United States; ^4^Department of Biomedical Sciences, Infectious and Immunological Diseases Research Center (IIDRC), Cedars-Sinai Medical Center, Los Angeles, CA, United States; ^5^David Geffen School of Medicine at University of California Los Angeles (UCLA), Los Angeles, CA, United States; ^6^Department of Pathology and Laboratory Medicine, Cedars-Sinai Medical Center, Los Angeles, CA, United States; ^7^Systemic Autoimmunity Branch, National Institute of Arthritis and Musculoskeletal and Skin Diseases, National Institutes of Health, Bethesda, MD, United States

**Keywords:** SLE, Ogg1, 8-OH-dG, cGAS-STING pathway, IFN-stimulated genes

## Abstract

Systemic Lupus Erythematosus (SLE) is a chronic inflammatory autoimmune disease in which type I interferons (IFN) play a key role. The IFN response can be triggered when oxidized DNA engages the cytosolic DNA sensing platform cGAS-STING, but the repair mechanisms that modulate this process and govern disease progression are unclear. To gain insight into this biology, we interrogated the role of oxyguanine glycosylase 1 (OGG1), which repairs oxidized guanine 8-Oxo-2′-deoxyguanosine (8-OH-dG), in the pristane-induced mouse model of SLE. *Ogg1*^−/−^ mice showed increased influx of Ly6C^hi^ monocytes into the peritoneal cavity and enhanced IFN-driven gene expression in response to short-term exposure to pristane. Loss of *Ogg1* was associated with increased auto-antibodies (anti-dsDNA and anti-RNP), higher total IgG, and expression of interferon stimulated genes (ISG) to longer exposure to pristane, accompanied by aggravated skin pathology such as hair loss, thicker epidermis, and increased deposition of IgG in skin lesions. Supporting a role for type I IFNs in this model, skin lesions of *Ogg1*^−/−^ mice had significantly higher expression of type I IFN genes (*Isg15, Irf9*, and *Ifnb*). In keeping with loss of *Ogg1* resulting in dysregulated IFN responses, enhanced basal and cGAMP-dependent *Ifnb* expression was observed in BMDMs from *Ogg1*^−/−^ mice. Use of the STING inhibitor, H151, reduced both basal and cGAMP-driven increases, indicating that OGG1 regulates *Ifnb* expression through the cGAS-STING pathway. Finally, in support for a role for OGG1 in the pathology of cutaneous disease, reduced *OGG1* expression in monocytes associated with skin involvement in SLE patients and the expression of *OGG1* was significantly lower in lesional skin compared with non-lesional skin in patients with Discoid Lupus. Taken together, these data support an important role for OGG1 in protecting against IFN production and SLE skin disease.

## Introduction

SLE, a chronic systemic inflammatory autoimmune disease, occurs in about 1.8–7.6 people per 100,000 in the United States, with a 9:1 female to male ratio (1). Patients with SLE have a diverse range of immunological abnormalities that contribute to disease progression and pathology. Despite the heterogeneity of the disease, type I interferons (IFN) have emerged as key pathogenic cytokines in SLE and correlate with disease severity (2, 3). One source of extracellular DNA that initiates and drives type I IFN production is genomic DNA released from dead and dying cells, which is recognized by TLR9 in endosomes, driving IFNα expression. More recently, oxidized DNA from mitochondria (driven by ROS production) has also been recognized as a trigger for type I IFN production. In SLE, low density neutrophils/granulocytes are an important source of oxidized mitochondrial DNA (mtDNA), which is released from activated neutrophils as they undergo NETosis. Indeed, NETs are enriched in oxidized mtDNA, specifically 8-OH-dG motifs (4, 5). In addition, exposure to ultravliolet light can also drive oxidative stress, oxidized DNA damage, and accumulation of 8-OH-dG lesions in the skin. Oxidized DNA, including 8-OH-dG, is sensed by the cytosolic DNA sensor cyclic GMP-AMP synthase (cGAS), which has recently emerged as an important contributor to elevated type I IFN in SLE (6, 7). Once activated, cGAS generates 2′3′-cyclic-dinucleotides which bind and activate the ER-resident adaptor protein stimulator of IFN genes (STING), and drive TBK1-IRF3 dependent induction of IFNβ (7–10).

Mechanisms regulating clearance of cytosolic DNA and RNA, including oxidized and damaged DNA, are critical to preventing triggering of IFN responses. OGG1 (oxoguanisine glycolase 1) is a DNA repair enzyme in the base excision repair (BER) pathway that excises and repairs 8-OH-dG DNA lesions (11). A single nucleotide polymorphism (SNP) in *OGG1* is associated with development of lupus nephritis (12). This SNP encodes a serine-to-cysteine substitution at position 326 (S326C), which is thought to reduce function of OGG1 (13), thus conferring susceptibility to lupus nephritis by enabling accumulation of 8-OH-dG (4, 14–16).

Given that OGG1 plays an important role in protecting against accumulation of oxidized DNA lesions, we hypothesized that loss of *Ogg1* may contribute to IFN-driven disease. We therefore investigated the role of OGG1 in the pristane-induced lupus (PIL) mouse model of IFN-driven SLE, which recapitulates numerous human SLE manifestations, including elevated type I IFNs, autoantibody generation, arthritis, and severe glomerulonephritis (17, 18). In this study, loss of *Ogg1* resulted in increased oxidized DNA damage, enhanced recruitment of Ly6C^hi^ monocytes and ISG expression in the peritoneal cavity, enhanced neutrophil activity, and systemic IFN-driven responses. Intriguingly, however, rather than increased kidney pathology, loss of *Ogg1* resulted in cutaneous involvement in PIL, accompanied by enhanced IFN driven gene expression in the skin. Mechanistically, we observed enhanced signaling through the cGAS-STING pathway in *Ogg1*^−/−^ bone marrow derived macrophages (BMDMs), an effect inhibited completely by blocking signaling through STING. In translating these results to human disease, we found decreased *OGG1* expression in SLE patients associated with cutaneous involvement, and that lesional skin from patients with chronic cutaneous lupus erythematosus had reduced *OGG1* expression compared to non-lesional areas. Taken together, our results indicate that OGG1 protects against IFN induction and cutaneous involvement in SLE by reducing 8-OH-dG driven IFN responses.

## Materials and Methods

### Mice

Wild-type C57BL/6 and *Ogg1*^−/−^ mice (C57BL/6 background), female, 6–8 weeks old, received a single i.p. injection of 0.5 ml of pristane (2,6,10,14-Tetramethylpentadecane (TMPD), Sigma). Some mice were sacrificed at 1 week after pristane. Mice were bled at 7 months after pristane inoculation for complete blood cell count analysis. All mice were monitored for proteinuria by dipstick test once a month and sacrificed at 10 months after pristane treatment.

### Flow Cytometry

The following conjugated anti-mouse antibodies were used: anti-Ly-6G (1A8), anti-CD11b (M1/70), anti-Ly-6C (ER-MP20), anti-F4/80 (BM8), anti-CD4 (GK1.5), anti-CD8a (53–6.7), anti-CD11c (3.9), and anti-B220 (RA3-6B2) (eBiosciences). Cells were incubated in CD16/32 (Fc block; BD Biosciences) prior to staining. Cells were acquired on LSR II (BD Biosciences) and analyzed with the FlowJo software (Treestar).

### Autoantibody and Cytokine ELISAs

Serum IgG subtypes (IgG1 and IgG2a) were measured by ELISA using the following coating antibodies: purified anti-mouse IgG1 and IgG2a and detection antibodies: biotin anti-mouse IgG1 and IgG2a (BD Pharmingen, San Jose, CA). Following Streptavidin-HRP (Sigma) incubation, the ELISA was developed with TMB substrate (Dako). The optical density (OD) for each well with a microplate reader set to 450 nm. MCP-1 and s100a8/9 levels were measured via ELISA according to the manufacturer's instruction (R&D). Total serum IgG and anti-dsDNA and anti-RNP Ab levels were quantified by ELISA using commercially available kits (Alpha Diagnostic International, San Antonio, TX), following manufacturer's protocols. An anti-mouse albumin ELISA kit was used to measure urine proteinuria (Bethyl labs).

### Gene Expression Analysis

Quantitative PCR (Q-PCR) was performed as previously described (19, 20). In brief, total RNA was extracted from 10^6^ peritoneal cells using TRIzol reagent (Invitrogen, Carlsbad, CA), and cDNA was synthesized using the (Invitrogen) according to the manufacturer's protocol. qPCR was performed on a CFX96 Real-Time PCR Detection System (Biorad). A melting-curve analysis was performed to ensure specificity of the products. Gene expression was normalized to GAPDH, and expression relative to the PBS treatment was calculated using the ddCt method (18) Primer sequences are listed as follows:

m*Isg15* Fw: GGTGTCCGTGACTAACTCCAT
m*Isg15* Rv: CTGTACCACTAGCATCACTGTG
m*Mx1* Fw: GATCCGACTTCACTTCCAGATGG
m*Mx1* Rv: CATCTCAGTGGTAGTCAACCC
m*Irf9* Fw: TGTCTGGAAGACTCGCCTAC
m*Irf9* Rv: GCAACATCCATACGACCTCTCT
m*Ifnb* Fw: CAGCTCCAAGAAAGGACGAAC
m*Ifnb* Rv: GGCAGTGTAACTCTTCTGCAT
m*Ogg1* Fw: tgtgtaccgaggagacgaca
m*Ogg1* Rv: ctgtgccaggctgacatcta
h*OGG1* Fw: CACACTGGAGTGGTGTACTAGC
h*OGG1* Rv: CCAGGGTAACATCTAGCTGGAA

### NETosis

NETosis was measured with or without stimulation with the ionophore A23187 for 0, 60, and 120 min in bone marrow derived neutrophils (BMDN). Sytox (ST) measures external DNA and Pico Green (PG) measures total DNA and ratio ST/PG was calculated.

### Histology

Skin samples from the shaved dorsal neck region were fixed in 10% buffered formalin and embedded in paraffin. Sections were stained with hematoxylin and eosin. Frozen sections were prepared and stained with anti-mouse IgG-AF550 conjugated antibody. Kidneys were fixed in paraformaldehyde, embedded in paraffin, and sections were stained with H&E and periodic acid-Schiff (PAS). Kidney sections were scored by pathologist in a blinded fashion to determine a glomerular and interstitial inflammation score. Briefly, a mean glomerular score was calculated for each mouse by grading injury in fifty glomeruli. Glomeruli were scored as normal = 0, mesangial expansion =1, endocapillary proliferation = 2, capillaritis or necrotic changes = 3, or crescents = 4. The interstitial score was determined by examining 50 high power fields and scoring the interstitial inflammation on a scale from 0 to 4 as absent, involving <25%, 25–50%, or >50% of the interstitium. For immunofluorescence, sections were prepared from frozen kidneys in OCT (TissueTek). Glomerular immune complexes in kidney sections were detected by staining with Dylight 488 labeled goat anti-mouse IgG (minimal x-reactivity, Biolegend). Using the image analysis software, deposition of IC was scored by pathologist.

### Skin Disease Incidence

Cohorts of WT and *Ogg1*^−/−^ mice were followed over time, and the onset of macroscopic skin disease was recorded along with the ages of the animals. Mice were considered affected when an area >0.5 cm of hair loss and ulceration was noted.

### Animal Studies

All animal experiments were performed according to the guidelines and approved protocols (IACUC #8299) of the Cedars-Sinai Medical Center Institutional Animal Care and Use Committee. Cedars-Sinai Medical Center is fully accredited by the Association for Assessment and Accreditation of Laboratory Animal Care (AAALAC International) and abides by all applicable laws governing the use of laboratory animals. Laboratory animals are maintained in accordance with the applicable portions of the Animal Welfare Act and the guidelines prescribed in the DHHS publication, Guide for the Care, and Use of Laboratory Animals.

### Study Approval

#### Patient Samples

As described previously (21), all SLE patients (as per ACR diagnostic criteria) were recruited from Cedars-Sinai Medical Center, CA, USA. Age- and sex-matched healthy donors who had no history of autoimmune diseases or treatment with immunosuppressive agents were included. All participants provided informed written consent and the study received prior approval from the institutional ethics review board.

#### Isolation of PBMCs and Cellular Subsets

Peripheral blood mononuclear cells (PBMCs) were separated from whole blood by density-gradient centrifugation with Ficoll-Paque Plus (GE Healthcare). CD14^+^ monocytes were purified from fresh PBMCs by positive selection using magnetic CD14^+^ beads (Miltenyi Biotec) according to manufacturer's protocol.

### Statistics

All data are expressed as means ± SD. Statistical differences were measured using either an unpaired Student's *t*-test or 2-way analysis of variance (ANOVA) when appropriate with Bonferroni *post-hoc* test. Normality of data was assessed via a Shapiro–Wilk normality test. When the data analyzed was not distributed normally, we used the Mann–Whitney test or Kruskal–Wallis 1-way ANOVA with Dunn's *post-hoc* test. Data analysis was performed using Prism software version 7.0a (GraphPad, San Diego, CA). A *P*-value of < 0.05 was considered statistically significant. Asterisks in the figures represent the following: ^*^P < 0.05; ^**^P < 0.01; ^***^P < 0.001, and ^****^P < 0.0001.

## Results

### *Ogg1* Deficiency Exacerbates Pristane-Induced Lupus-Like Systemic Inflammatory Responses *in vivo*

**Figure 1:**
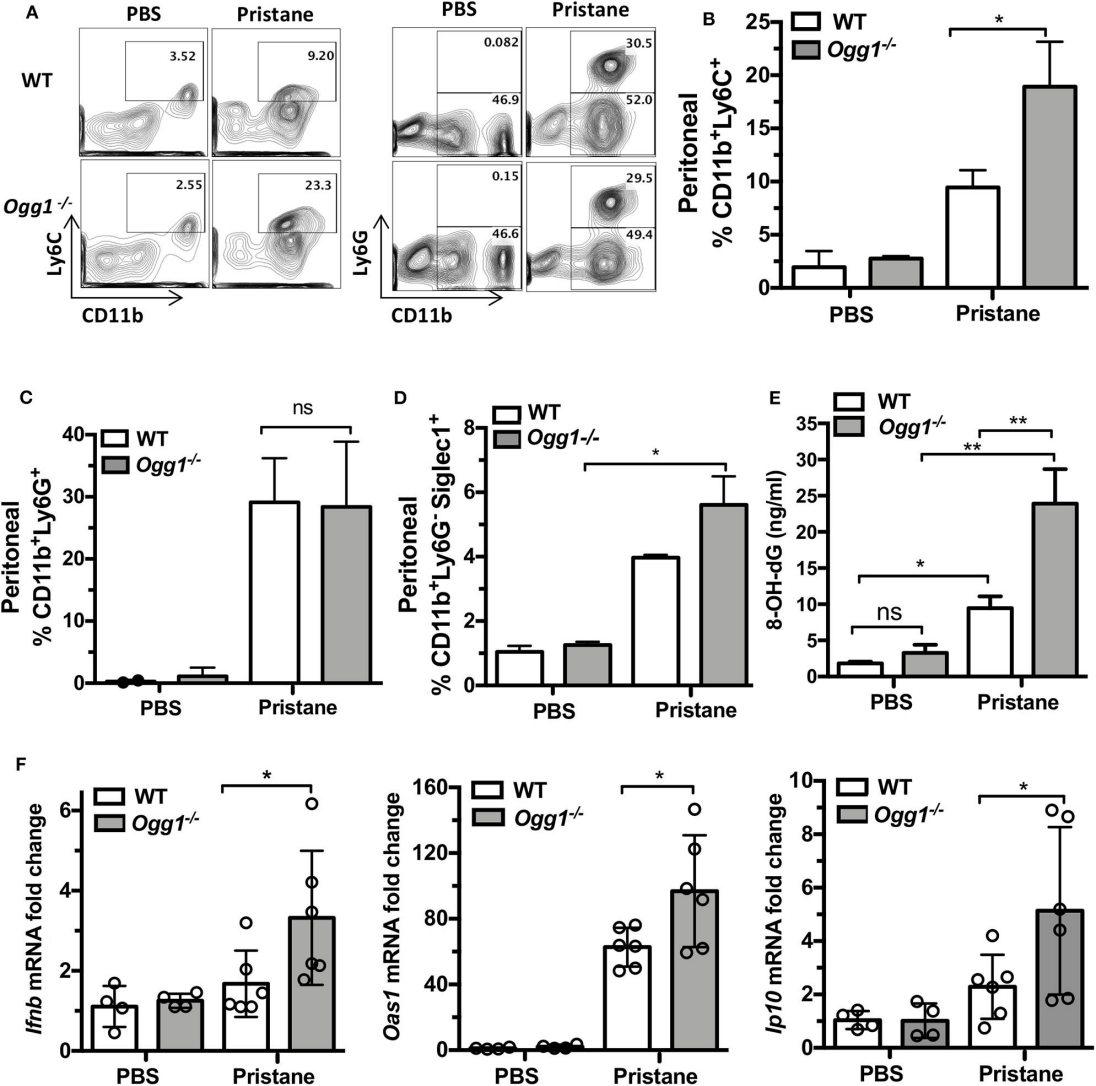
Early inflammatory response to pristane in WT and *Ogg1*^−/−^mice. **(A)** Representative pictures of FACS analysis of peritoneal cells day 7 after pristane. **(B)** % CD11bLy6C+ population, **(C)** % CD11bLy6G^+^ population, **(D)** Siglec1+ cells in CD11bLy6G^−^ population. All data represent mean ± SD. **(E)** 8-OH-dG ELISA peritoneal lavage. **(F)** The expression of mRNA for IFN signature genes (*Ifnb, Oas1*, and *Ip10*) in peritoneal cells. Statistical significance was determined using Two-Way ANOVA with Bonferroni *post-hoc* test. *p < 0.05, **p < 0.01.

To determine whether loss of *Ogg1* exacerbated disease progression in PIL, we compared C57Bl/6 (WT) and *Ogg1*^−/−^ mice 7 days after pristane treatment. Analysis of peritoneal lavage demonstrated that pristane treatment resulted in increased recruitment of inflammatory CD11b^+^Ly6C^hi^ monocytes and expression of Siglec1, an IFN inducible gene, in *Ogg1*^−/−^ mice compared to WT mice, whereas neutrophil (CD11b^+^Ly6G^+^) recruitment was similar across both genotypes (Figures 1A–D). In addition, significantly higher level of 8-OH-dG was determined in peritoneal lavage from *Ogg1*^−/−^ mice compared to WT mice (Figure 1E). Taken together, lack of *Ogg1* resulted in enhanced expression of IFN stimulated genes (ISGs)—*Ifnb, Oas1*, and *Ip10* (Figure 1F).

**Figure 2:**
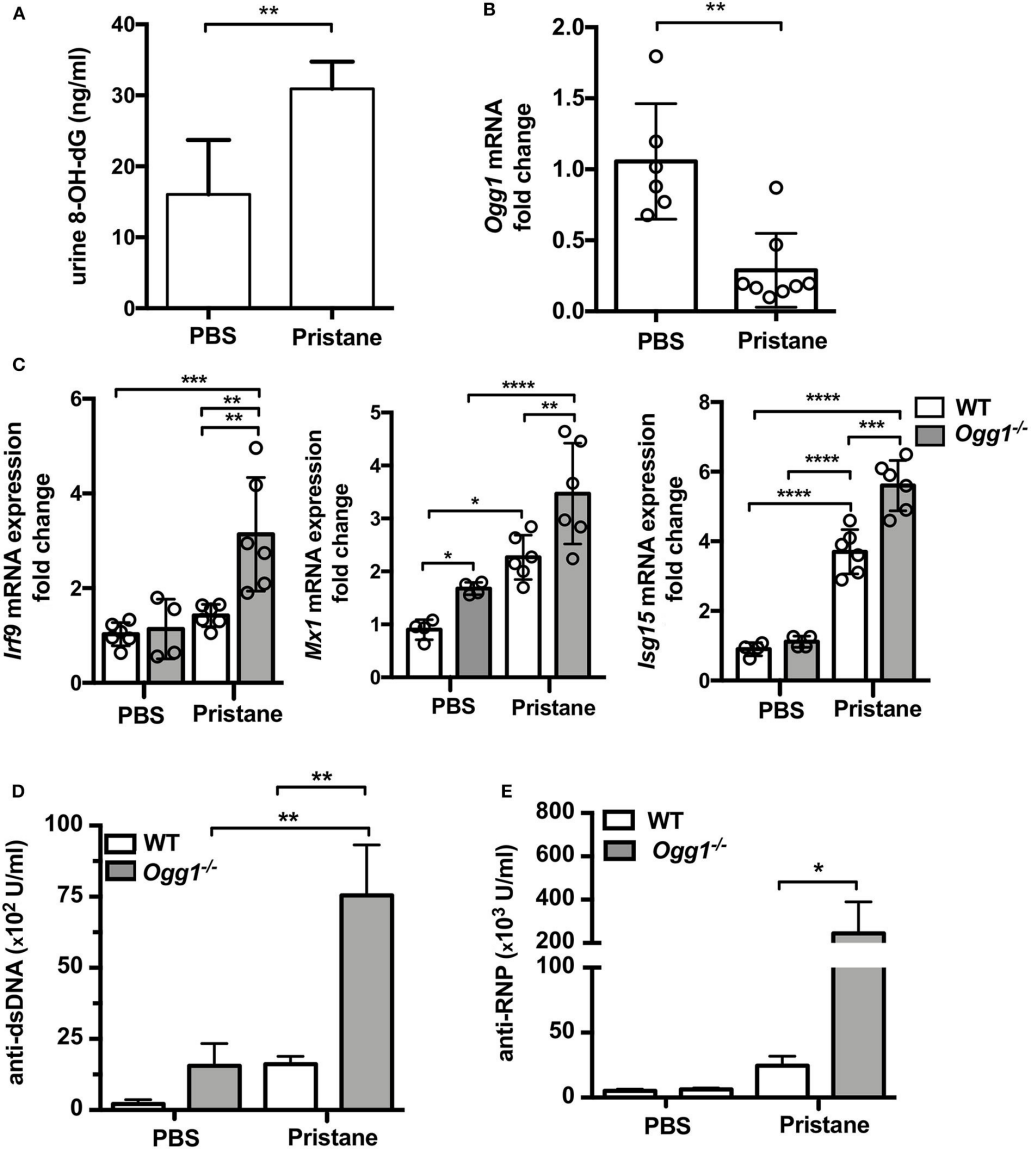
Increased inflammatory response to pristane in *Ogg1*^−/−^ mice. **(A)** 8-OH-dG secretion in spot urine in WT mice with or without pristane. *n* = 3–4/group **(B)** *Ogg1* mRNA expression in peritoneal cells in WT mice with or without pristane. *n* = 6–8/group **(C)** The expression of mRNA for IFN signature genes (*Irf9, Mx1*, and *Isg15*) in peritoneal cells. **(D,E)** The level of auto antibody, anti-dsDNA, and anti-RNP, in serum by ELISA. *n* = 10–15/group. All data represent mean ± SD. Statistical significance was determined using Two-way ANOVA with Bonferroni *post-hoc* test. *p < 0.05, **p < 0.01, ***p < 0.001, ****p < 0.0001.

**Figure 3:**
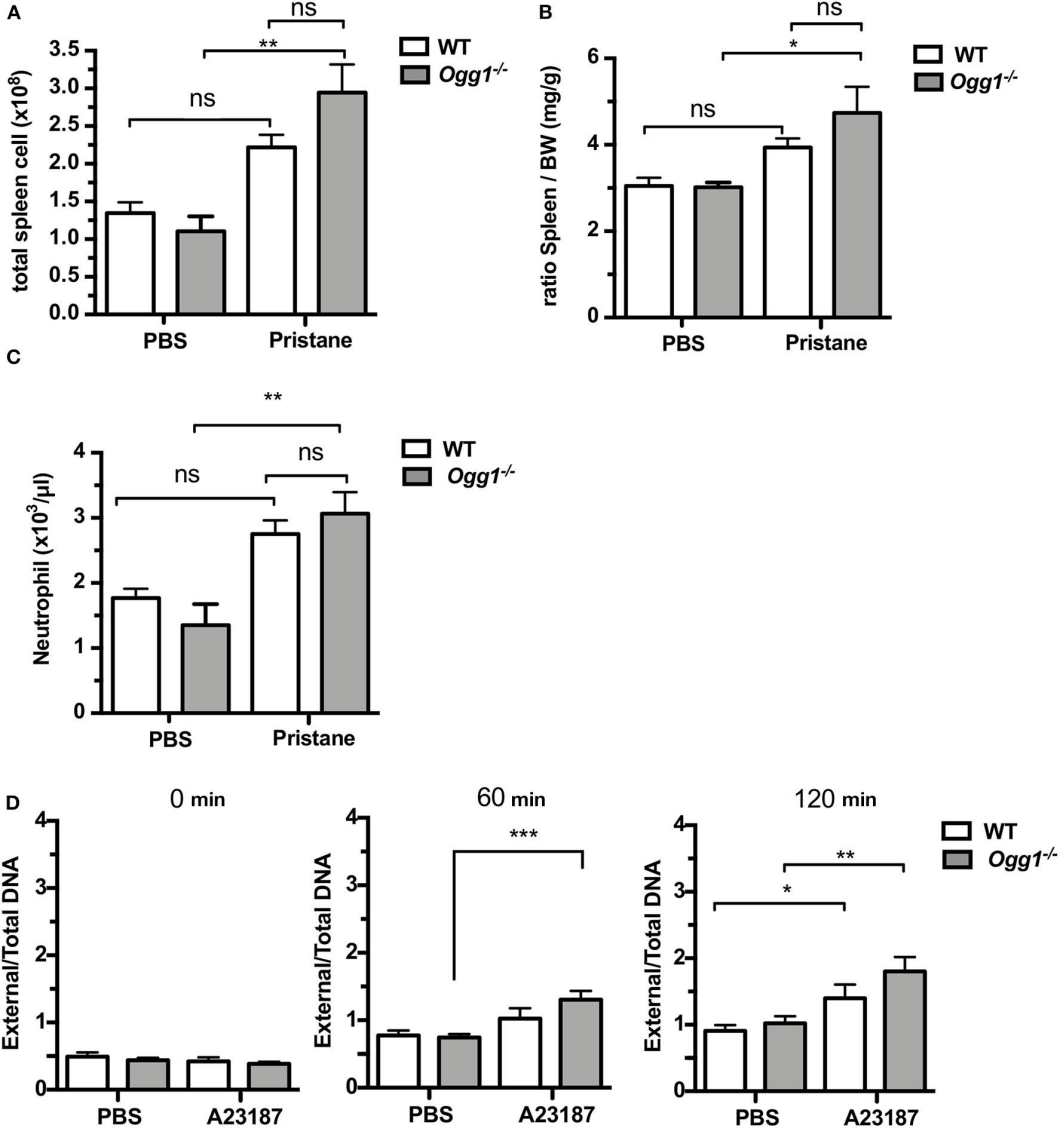
Increased inflammatory response to pristane in *Ogg1*^−/−^mice. **(A,B)** ratio of spleen to BW and Total cell number of spleen. **(C)** neutrophil counts by complete blood cell count (CBCC) analysis in peripheral blood. *n* = 4–6 per group **(D)** NETosis in A23187 stimulated BMDN obtained from pristane treated WT and *Ogg1*^−/−^ mice. *n* = 4 in triplicates. All data represent mean ± SD. Statistical significance was determined using Two-Way ANOVA with Bonferroni *post-hoc* test. ns, not significant. *p < 0.05, **p < 0.01, ***p < 0.001.

To determine the role of OGG1 in organ damage and SLE pathogenesis, we compared C57Bl/6 (WT) to *Ogg1*^−/−^ mice 10 months after pristane injection. In female C57Bl/6 mice, pristane treatment significantly elevated the level of 8-OH-dG in urine 10 months after injection, indicating that pristane induced oxidative DNA damage (Figure 2A). Furthermore, *Ogg1* expression was significantly lower in peritoneal cells from pristane-treated mice than untreated mice (Figure 2B), indicating that *Ogg1* downregulation may permit the accumulation of oxidative DNA damage in PIL mice. In keeping with our analysis of the acute effects of pristane on IFN-inducible responses in *Ogg1*^−/−^ mice we observed that expression of IFN stimulated genes (*Irf9, Mx1*, and *Isg15*) were significantly higher in the peritoneal cells of *Ogg1*^−/−^mice post pristane injection than in those of WT mice (Figure 2C), indicating a sustained systemic response to pristane treatment in the *Ogg1*^−/−^ mice. In order to assess the level of lupus-like systemic inflammatory responses induced by pristane, we measured circulating levels of anti-dsDNA and anti-RNP autoantibodies, and found that all were substantially higher in *Ogg1*^−/−^ mice than WT mice 10 months after pristane exposure (Figures 2D,E), whereas total IgG, IgG1, and IgG2a and other autoantibodies showed no difference between genotypes (Supplementary Figures 1A–D). Rather surprisingly whilst both pristane-treated WT and *Ogg1*^−/−^ mice exhibited splenomegaly 10 months post-pristane treatment compared with untreated mice, with no statistically significant difference observed between treated WT or *Ogg1*^−/−^ groups (Figure 3A). The splenomegaly observed was mirrored by a significant increase in the number of splenocytes (Figure 3B) and in circulating neutrophil numbers (as determined by complete blood count, Figure 3C and Supplementary Figure 2C) in pristane-injected WT and *Ogg1*^−/−^ mice, again with no statistically significant difference observed between treated WT or *Ogg1*^−/−^ groups. Similar changes were observed with respect to neutrophils (CD11b^+^Ly6G^+^) and inflammatory monocytes (CD11b^+^Ly6C^hi^) recruited to the peritoneal cavity following pristane treatment of both genotypes (Supplementary Figures 2A,B), whilst levels of S100 calcium binding protein A8/9 (s100a8/9, known as calprotectin), a potent chemotactic agent for neutrophils and monocytes both *in vitro* and *in vivo* (19), and MCP1 were similarly changed with no significant differences between treated groups (Supplementary Figures 2D,E). However, despite the lack of significant differences in absolute numbers between WT and *Ogg1* KO treated groups, functionally lack of *Ogg1* modified neutrophil function. Robust NET formation was observed following A23187 ionophore stimulation of bone-marrow derived neutrophils (BMDN) from both pristane-treated WT and *Ogg1*^−/−^mice. However, neutrophils from *Ogg1*^−/−^ mice displayed altered kinetics, with NET formation starting earlier and being more pronounced (although not significantly) in *Ogg1*^−/−^BMDN compared with WT BMDN (Figure 3D). Overall, these results suggest that lack of *Ogg1* leads to increased responses to 8-OH-dG, anti-dsDNA antibodies, and enhanced IFN responses in the pristane model.

### Loss of *Ogg1* Has Little Effect on Kidney Pathology, but Exacerbates Skin Pathology in Pristane-Treated Mice

To determine the effect of *Ogg1*-deficiency on glomerulonephritis in this model of lupus, we measured proteinuria. None of the mice from untreated or treated groups scored 3+, although *Ogg1*^−/−^mice treated with pristane showed a non-significant trend toward increased pathological score (Supplementary Figure 3A). IgG deposition within the glomeruli was enhanced in both pristane-treated WT and *Ogg1*^−/−^ mice compared with their untreated controls (Supplementary Figure 3B). We next scored kidney sections stained with PAS and Mesangial expansion/hypercellularity (M.E.) and Endocapillary Hypercellularity (H.E.) indices, which approximate the scoring system of human lupus nephritis. Both M.E and H.E were significantly increased in both pristane-treated WT and *Ogg1*^−/−^ mice compared with their control groups, with a non-significant trend toward increased pathology in *Ogg1*^−/−^ mice compared with WT (Supplementary Figure 3C). These results indicate that loss of *Ogg1* may only play a minor role in the development of pristane induced lupus-like glomerulonephritis.

**Figure 4:**
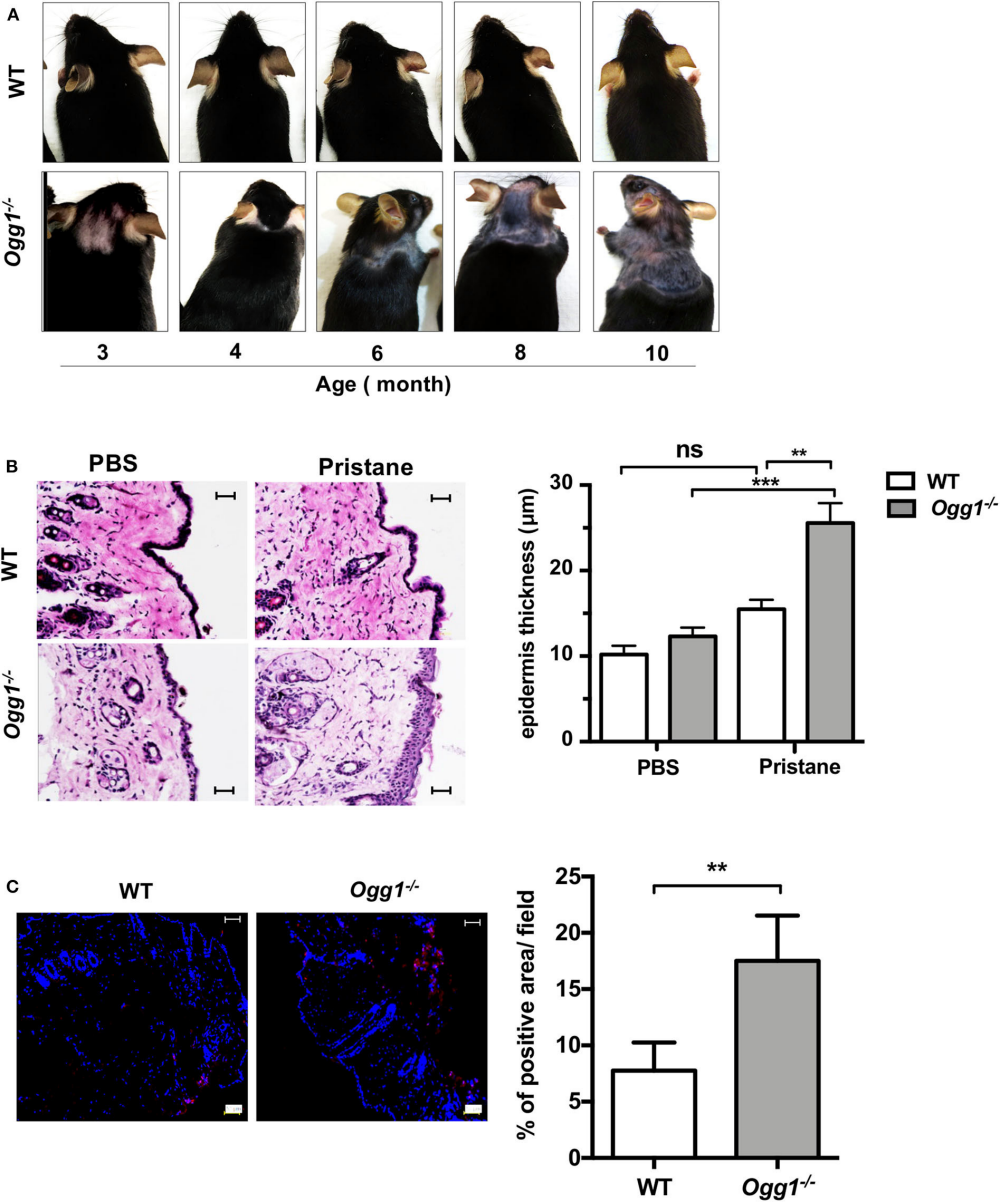
*Ogg1* deficiency exacerbates skin changes in pristane treated mice. **(A)** Representative pictures of hair loss observed over the indicated time periods in pristane treated WT and *Ogg1*^−/−^mice. **(B)** Representative pictures of H&E staining of skin from dorsal neck area. Quantification of epidermal thickness. Scale bar = 20 μm. *n* = 10–12/group **(C)** Representative pictures and quantification of IgG deposition of dorsal neck area of skin of pristane treated WT and *Ogg1*^−/−^. *n* = 4/group. All data represent mean ± SD. Statistical significance was determined using Two-Way ANOVA with Bonferroni *post-hoc* test in panel **(B)** and two tails Student's *t*-test in panel **(C)**. ns, not significant. **p < 0.01, ***p < 0.001.

Surprisingly, we observed increased hair loss and differences in skin pathology in the *Ogg1*^−/−^ compared to WT. Starting around 3–3.5 months of age, both untreated and treated *Ogg1*^−/−^ mice lost hair in the dorsal neck area, whereas WT mice did not. In pristane-treated *Ogg1*^−/−^ mice, hair loss spread to the frontal, and dorsal part of entire trunk, reaching the hind legs by 10 months of age (Figure 4A and Supplementary Figure 4A). Histopathological analysis of H&E stained sections revealed epidermal hyperplasia, consisting of acanthosis and hyperkeratosis, in pristane-treated *Ogg1*^−/−^ mice (Figure 4B). Analysis of IgG deposition in the skin from the dorsal neck area revealed that skin of *Ogg1*^−/−^ mice had visible IgG deposition, whereas WT had none (Figure 4C). These results indicate that *Ogg1* deficiency promotes the development of pristane-induced skin disease.

### Inhibition of STING Reverses Increased IFN Response in BMDMs From *Ogg1^−/−^* Mice

**Figure 5:**
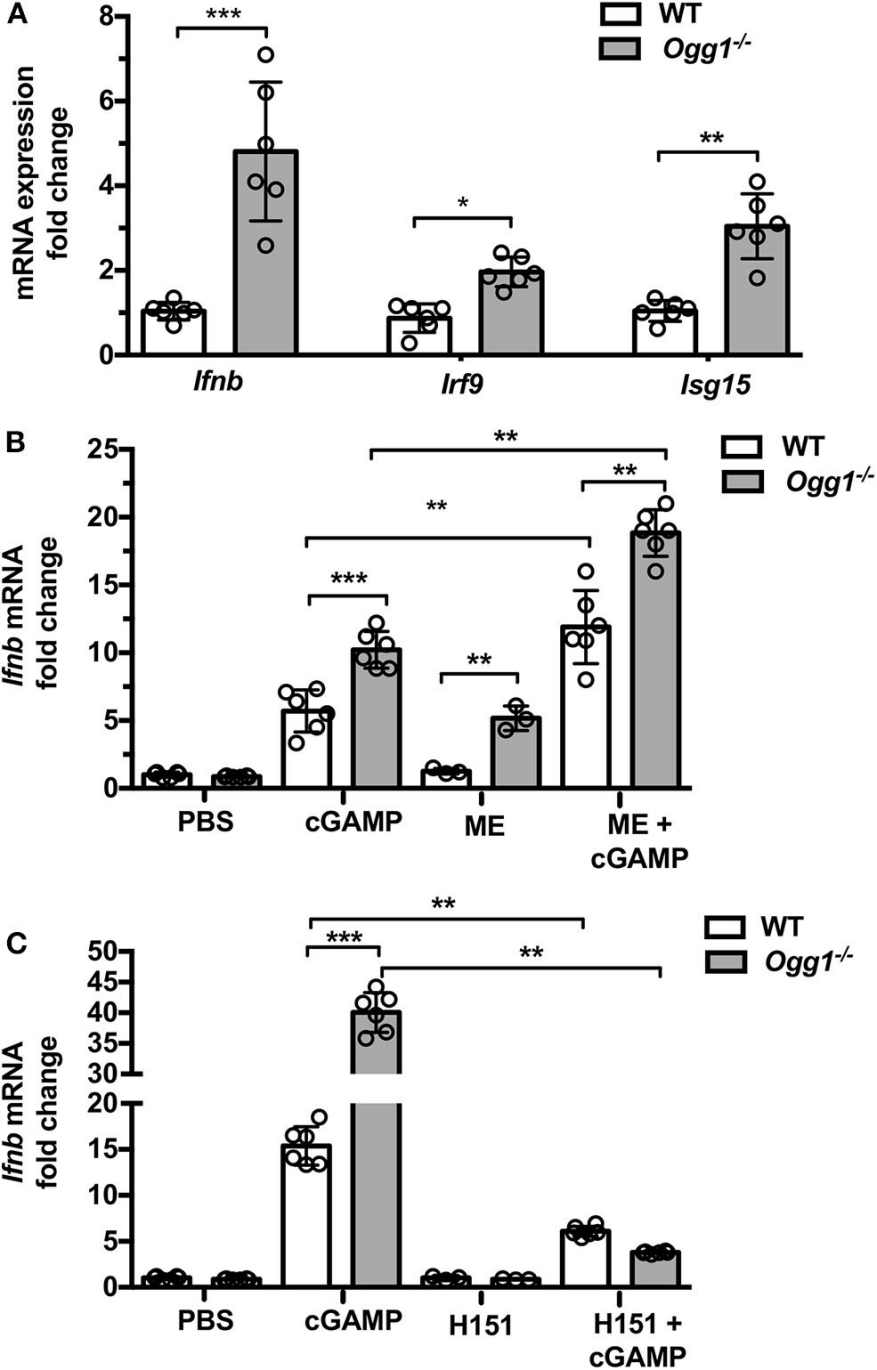
Loss of *Ogg1* results in enhanced STING-driven type I IFN response. **(A)** The expression of mRNA for IFN signature genes (*Ifnb, Irf9*, and *Isg15*) affected area of skin, fold changes were *Ogg1*^−/−^ WT. **(B)** BMDM from WT and *Ogg1*^−/−^mice, were stimulated with cGAMP with or without 50 μM menodione—ME (ROS inducer) pretreatment. *Ifnb* mRNA was measured by RT-PCR. **(C)** *Ifnb* mRNA in BMDM from WT and *Ogg1*^−/−^mice, were stimulated with cGAMP with or without H151 (STING inhibitor). *n* = 5–6 per group. All data represent mean ± SD. Statistical significance was determined using two tails Student's *t*-test **(A)** Two-way ANOVA with Bonferroni *post-hoc* test **(B,C)**. ^*^p < 0.05, ^**^p < 0.01, ^***^p < 0.001.

To assess whether type I IFN may play a role in skin pathology in the pristane-treated *Ogg1*^−/−^ mice, we measured IFN-stimulated gene expression in dorsal skin from pristane-treated mice. Pristane treatment induced significantly higher expression of *Ifnb* and type I IFN signature genes (*Irf9* and *Isg15*) in dorsal neck skin of *Ogg1*^−/−^mice than WT mice (Figure 5A). Given the recently reported role of the cGAS-STING pathway in both recognition of 8-OH-dG DNA lesions and cutaneous LE (20), we interrogated the role of STING downstream of 8-OH-dG recognition in *Ogg1*^−/−^ mice. Treatment of WT and *Ogg1*^−/−^ BMDMs with 50 μM menodione to induce 8-OH-dG (22), resulted in enhanced *Ifnb* expression in *Ogg1*^−/−^ BMDMs compared to controls (Figure 5B). Interestingly, menodione also amplified the response to cGAMP in *Ogg1*^−/−^ BMDMs, indicating that loss of *Ogg1* primed the cGAS-STING pathway. Confirming a role for STING in the enhanced *Ifnb* expression observed in *Ogg1*^−/−^ BMDMs, the STING inhibitor H151 (1 μM), completely blocked menodione- and cGAMP-induced *Ifnb* expression in *Ogg1*^−/−^ BMDM (Figure 5C). As a control, we did not observe any changes in polyIC induced *Ifnb* expression in H151-pretreated cells compared to non-treated cells (Supplementary Figure 5). Taken together, these data indicated that the cGAS-STING pathway may be responsible for increased type I IFN expression in *Ogg1*^−/−^ cells.

### Altered Levels of *OGG1* Associate With Skin Disease in SLE Patients

**Figure 6:**
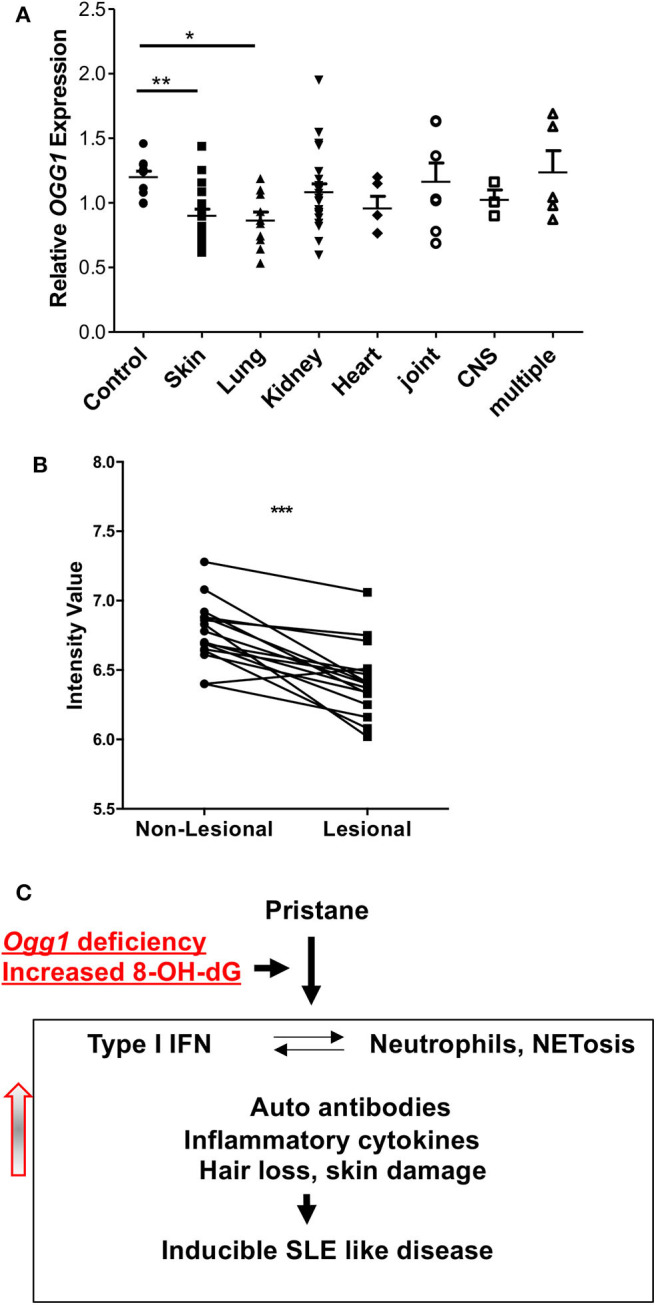
*OGG1* expression in human patients. **(A)** *OGG1* mRNA expression of PBMC of healthy control and SLE patients with different organ involvements. **(B)** *OGG1* expression from paired lesional and non-lesional skin samples (*n* = 16) from patients with Discoid Lupus Erythematosus (DLE) (Data available at NCBI GEO database, accession GSE100093). Values are reported as mRNA expression levels, i.e., normalized fluorescent intensity. Lines indicate relationship between paired samples while *** indicates the difference is significant between the two groups at *p* < 0.00001 (Paired Student's *t*-test). **(C)** The proposed mechanism for increased inflammatory response to pristane in due to the loss of OGG1. *p < 0.05, **p < 0.01.

To determine how our observations of cutaneous involvement in pristane-treated *Ogg1*^−/−^ mice translated to human SLE, we looked at potential clinical associations of *OGG1* expression in our SLE patient cohort (21). This cohort had been previously screened for increased expression of ISGs in peripheral blood mononuclear cells as shown in Smith et al. (21). Monocytes from these patients with skin and lung involvement expressed lower levels of *OGG1* than monocytes from healthy controls, whereas no such decrease was observed in monocytes from patients with other organ involvement (Figure 6A). Analysis of gene expression profiling conducted by Werth et al. (23) on paired skin samples from lesional and non-lesion skin from patients with chronic cutaneous (Discoid) Lupus Erythematosus (DLE) showed that expression of *OGG1* was significantly reduced in lesional skin compared with non-lesional skin (adj. *p* = 3.02 × 10^−3^; data accessible at NCBI GEO database (24, 25), accession GSE100093) (Figure 6B). Taken together, our data demonstrate that expression of *OGG1* in SLE is negatively associated with skin damage observed in patients. This suggests that reduced expression or loss of *OGG1* promotes inflammation and IFNβ production via recognition of oxidized DNA such as 8-OH-dG by the cGAS-STING pathway, contributing to both localized responses in the skin and systemic inflammation (Figure 6C).

## Discussion

In this study, we explored the contribution of OGG1 to the pathogenesis of pristane-induced lupus. Deficiency of *Ogg1* resulted in enhanced immune responses to pristane, namely increased recruitment of Ly6C^hi^ inflammatory monocytes to the peritoneal cavity, enhanced NETosis, systemic expression of type I IFN signature genes, higher levels of autoantibodies and skin damage. Gene expression analysis showed decreased *Ogg1* mRNA level in DLE lesions and in PBMCs from SLE patients with skin involvement. This demonstrates the importance of oxidized DNA repair enzyme OGG1 in protecting against oxidized DNA-induced IFN responses in lupus.

OGG1 has two isoforms: nuclear and mitochondrial (11). Multiple approaches have demonstrated that mitochondrial OGG1 is crucial for maintenance cell survival, mitochondrial function, and energetics (26, 27). Recognition and repair of 8-OH-dG lesions is carried out by the mitochondrial isoform, and we previously showed that *Ogg1*^−/−^ BMDMs were more sensitive to menadione-induced mtDNA oxidative damage, and that inflammation caused by oxidized DNA damage and dyslipidemia-induced metabolic stress resulted in accelerated atherosclerosis in *Ogg1*^−/−^*Ldlr*^−/−^ mice (22).

It is well-known that the catalytic activity of OGG1 is decreased in the setting of oxidative stress, resulting in an accumulation of 8-OH-dG (28). In turn, the accumulation of oxidized DNA drives both inflammation and induction of type I IFNs, which are thought to be the principal drivers of SLE. Our results show that *Ogg1* deficiency results in increased proinflammatory monocytes and neutrophils, which are the main source of type I IFN in the pristane model. Abnormal accumulation of self DNA or oxidized DNA in the cytosol engages the DNA sensor cGAS, and promotes STING-IRF3-dependent signaling to elevate interferon-stimulated gene expression, potentiating type I IFN responses (29, 30). Indeed, recent evidence points to STING-mediated pathways as being overactivated in SLE patients (31). Here, the results of STING activator and inhibitor experiments (Figure 5) demonstrate that STING is essential for an increased expression of type I IFN in *Ogg1*^−/−^ mice. During the past decade, significant progress has been made to elucidate a role for aberrant DNA repair in the development of lupus. Recent evidence indicates that patients with SLE have higher levels of DNA damage than normal subjects, and that polymorphisms in genes involved in the preservation of the genomic stability increase the risk for SLE (32–34). Furthermore, experience from animal models reinforces the importance of defective repair in the development of SLE-like disease (35), suggesting that therapeutic potential of targeting DNA damage and DNA repair responses in SLE pathogenesis (36, 37). Indeed, oxidized DNA has a greater immunostimulatory capacity to stimulate type I IFN than does normal DNA, possibly due to increased resistance to degradation by the 3′ repair exonuclease 1 TREX 1 (38). Our results show that the ROS inducer menodione more potently induces type 1 IFN expression from *Ogg1*^−/−^ BMDMs, in keeping with the model that lack of *Ogg1* results in accumulation of oxidized DNA, driving a more potent activation of the STING-dependent cytoplasmic DNA-sensing pathway.

There are 21 known polymorphisms in *OGG1*, 13 of which are associated with disease due to reduced capacity to repair DNA damage (13, 39, 40). In addition, *OGG1* polymorphisms have been linked to SLE risk. For example, the *OGG1* SNP rs1052133 is associated with the development of lupus nephritis and an observed increase of 8-OH-dG levels in plasma (12). Interestingly, however, we observed no exacerbated kidney pathology in the *Ogg1*^−/−^ mice in response to pristane treatment. Instead, we find that loss of *Ogg1* exacerbates pristane-induced skin pathology and increased expression of IFN stimulated genes in the skin of *Ogg1*^−/−^ mice. Although the exact mechanism of skin involvement in SLE is unknown, ultraviolet light (UV), immune cells, cytokines, and deposition of immunoglobulins are all known to have a role in the development of skin inflammation in SLE (41–43). Recent studies also suggest a pathogenic role of endogenous nucleic acids in SLE with cutaneous involvement (44, 45). 8-OH-dG is abundant in skin lesions in SLE patients with cutaneous involvement, and colocalizes with an IFN gene signature (44, 46), suggesting that oxidized DNA damage is an important driver of pathology. In support of this finding, injection of oxidized DNA into the skin of lupus-prone MRL/Ipr mice induces lesions similar to those observed in patients (38). In keeping with these observations and consistent with our findings in the *Ogg1*^−/−^ mice, we find that *OGG1* expression is reduced in SLE patients with skin involvement and that *OGG1* expression is decreased in skin lesions obtained from DLE patients compared to non-lesional matched samples. Taken together, our results indicate that OGG1 protects against IFN induction and cutaneous involvement in SLE by reducing 8-OH-dG-driven IFN reponses, via a mechanism that involves cGAS-STING mediated IFNβ production.

## Data Availability Statement

All datasets generated for this study are included in the article/Supplementary Material.

## Ethics Statement

The studies involving human participants were reviewed and approved by Cedars Sinai ethics review board. The patients/participants provided their written informed consent to participate in this study. The animal study was reviewed and approved by Cedars-Sinai Medical Center Institutional Animal Care and Use Committee.

## Author Contributions

GT, SC, MJK, KS, and TRC designed the experiments and conceived the overall research plan. GT, ENM, DEL, GD, JY, ML, LPB, JLM, and MY conducted the experiments and analyzed results. DJW and MI enrolled patients. GT and CAJ wrote the manuscript. All authors contributed to manuscript preparation. CAJ and MA contributed to supervision of the work.

## Conflict of Interest

The authors declare that the research was conducted in the absence of any commercial or financial relationships that could be construed as a potential conflict of interest.
