# Supplementary material for: Oxidative DNA Damage Accelerates Skin Inflammation in Pristane-Induced Lupus Model
Source: Front Immunol. 2020 Sep 24;11:554725. doi: 10.3389/fimmu.2020.554725 (PMC7541920; doi:10.3389/fimmu.2020.554725)
Supplement: Supplementary file 1 [file Presentation_1.PPTX]

## Slide 1
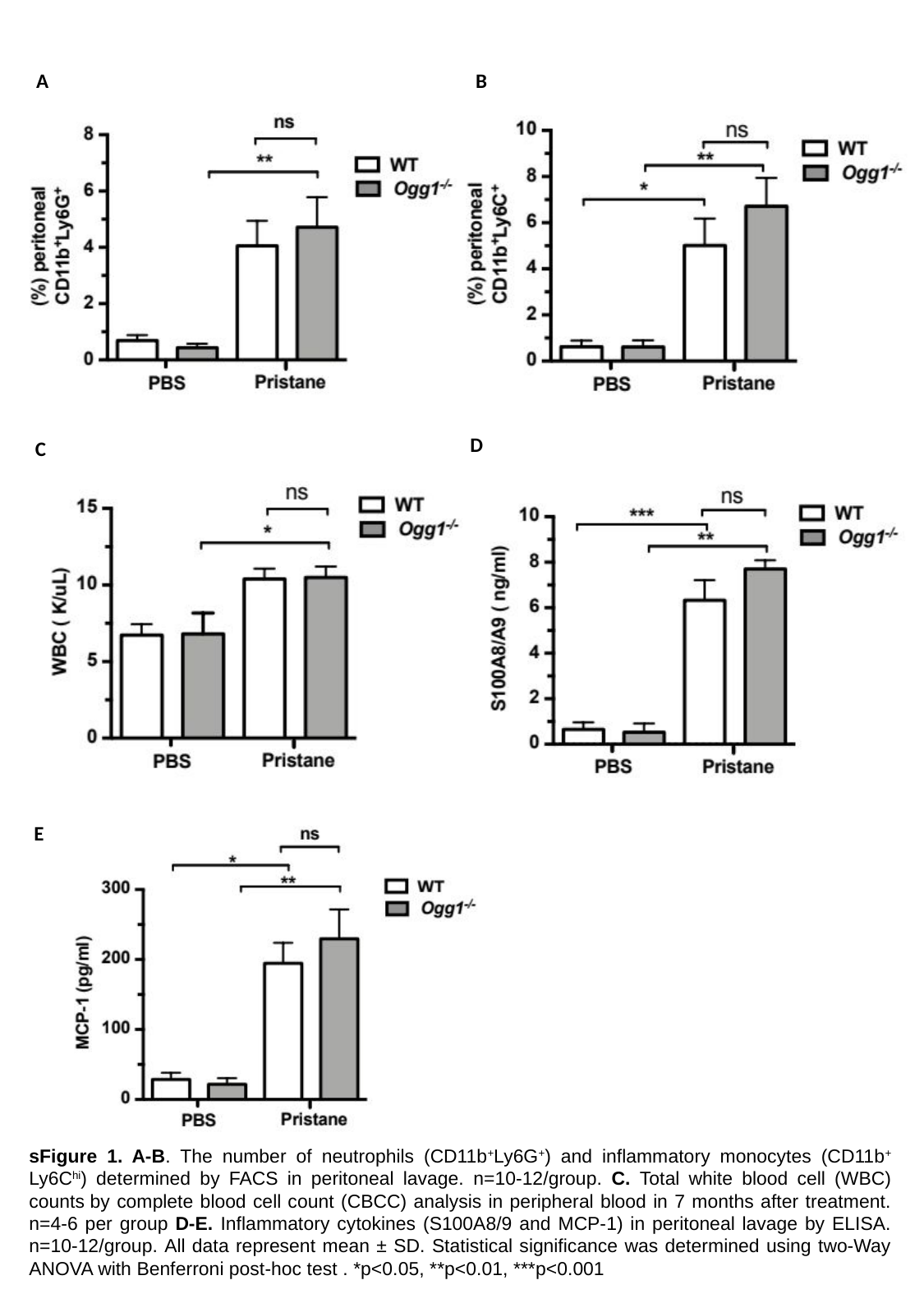

B
A
D
C
E
sFigure 1. A-B. The number of neutrophils (CD11b+Ly6G+) and inflammatory monocytes (CD11b+ Ly6Chi) determined by FACS in peritoneal lavage. n=10-12/group. C. Total white blood cell (WBC) counts by complete blood cell count (CBCC) analysis in peripheral blood in 7 months after treatment. n=4-6 per group D-E. Inflammatory cytokines (S100A8/9 and MCP-1) in peritoneal lavage by ELISA. n=10-12/group. All data represent mean ± SD. Statistical significance was determined using two-Way ANOVA with Benferroni post-hoc test . *p<0.05, **p<0.01, ***p<0.001

## Slide 2
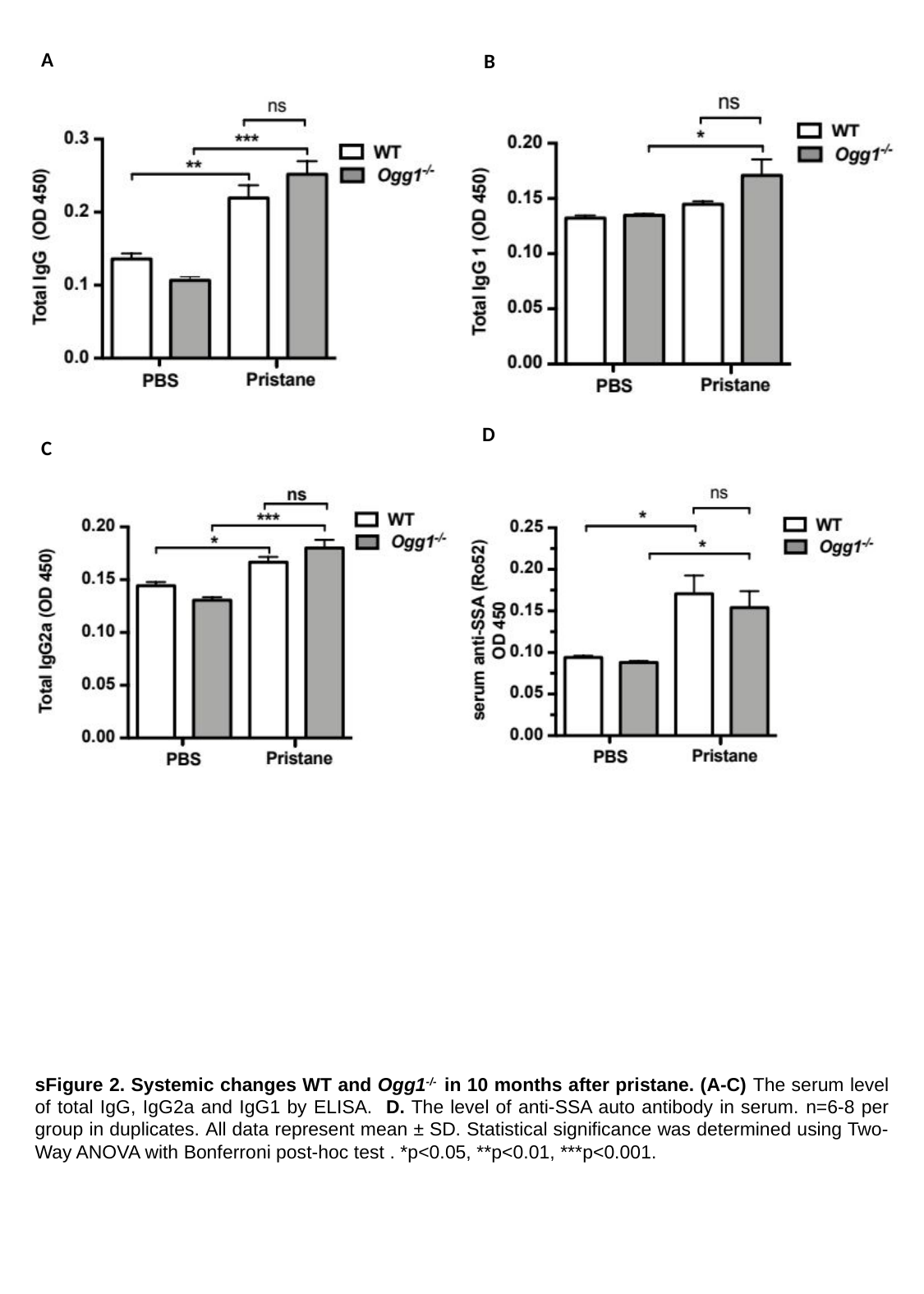

A
B
D
C
sFigure 2. Systemic changes WT and Ogg1-/- in 10 months after pristane. (A-C) The serum level of total IgG, IgG2a and IgG1 by ELISA. D. The level of anti-SSA auto antibody in serum. n=6-8 per group in duplicates. All data represent mean ± SD. Statistical significance was determined using Two-Way ANOVA with Bonferroni post-hoc test . *p<0.05, **p<0.01, ***p<0.001.

## Slide 3
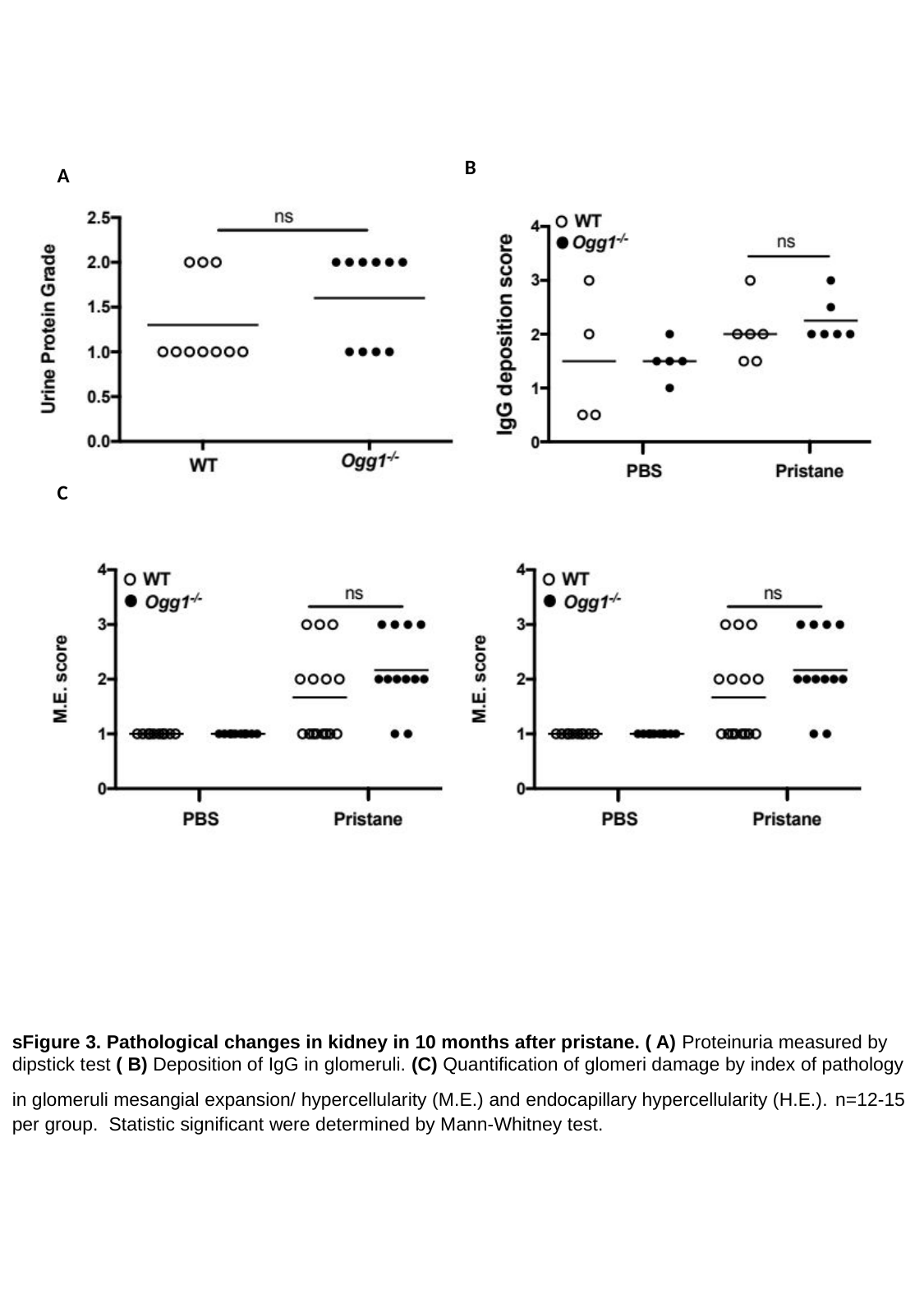

B
A
C
sFigure 3. Pathological changes in kidney in 10 months after pristane. ( A) Proteinuria measured by dipstick test ( B) Deposition of IgG in glomeruli. (C) Quantification of glomeri damage by index of pathology in glomeruli mesangial expansion/ hypercellularity (M.E.) and endocapillary hypercellularity (H.E.). n=12-15 per group. Statistic significant were determined by Mann-Whitney test.

## Slide 4
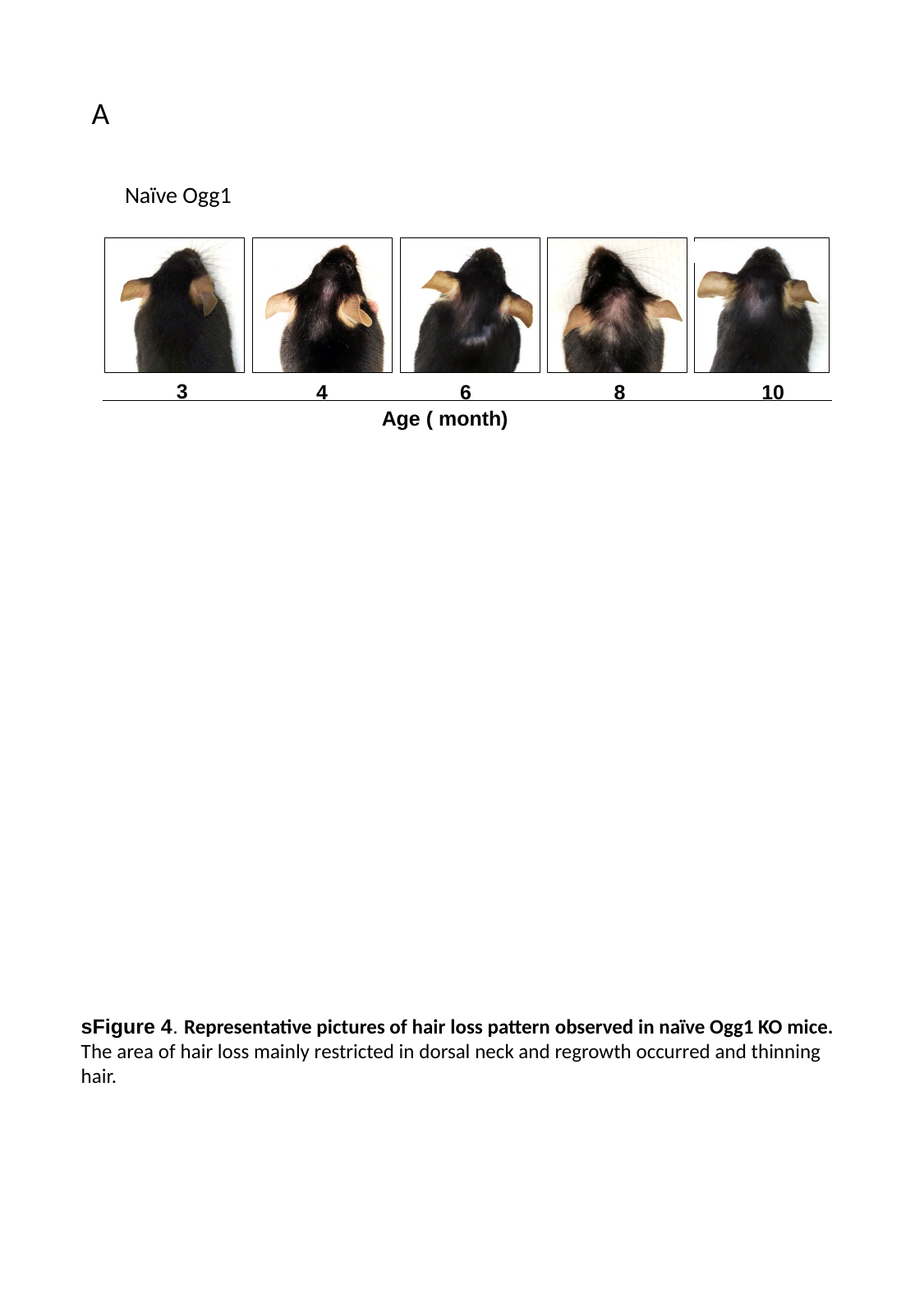

A
Naïve Ogg1
 3
4
6
8
10
Age ( month)
sFigure 4. Representative pictures of hair loss pattern observed in naïve Ogg1 KO mice. The area of hair loss mainly restricted in dorsal neck and regrowth occurred and thinning hair.

## Slide 5
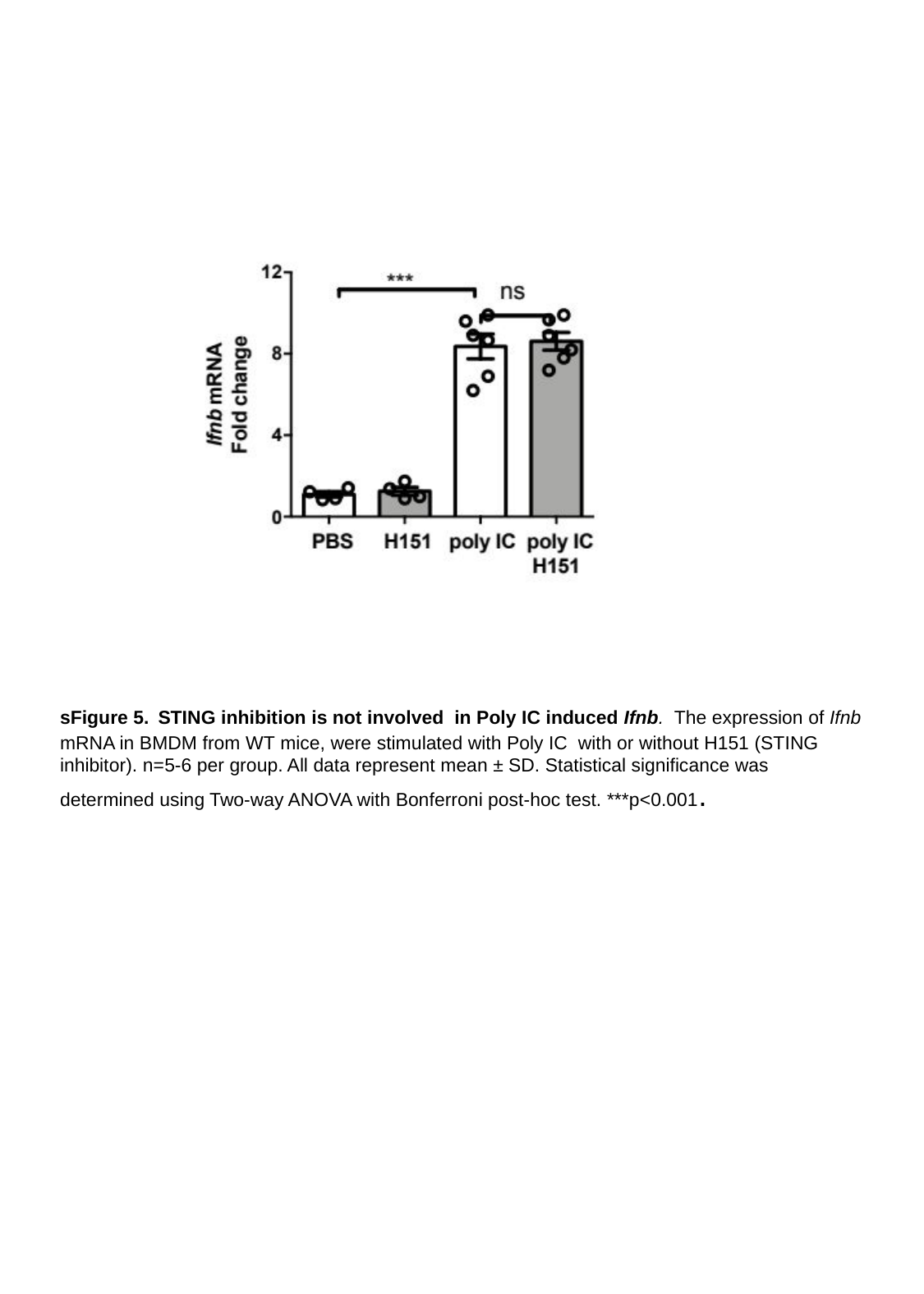

sFigure 5. STING inhibition is not involved in Poly IC induced Ifnb. The expression of Ifnb mRNA in BMDM from WT mice, were stimulated with Poly IC with or without H151 (STING inhibitor). n=5-6 per group. All data represent mean ± SD. Statistical significance was determined using Two-way ANOVA with Bonferroni post-hoc test. ***p<0.001.
